# Local control and patient reported outcomes after online MR guided stereotactic body radiotherapy of liver metastases

**DOI:** 10.3389/fonc.2022.1095633

**Published:** 2023-01-16

**Authors:** Laura Uder, Marcel Nachbar, Sarah Butzer, Jessica Boldt, Sabrina Baumeister, Michael Bitzer, Alfred Königsrainer, Thomas Seufferlein, Rüdiger Hoffmann, Sergios Gatidis, Konstantin Nikolaou, Daniel Zips, Daniela Thorwarth, Cihan Gani, Simon Boeke

**Affiliations:** ^1^ Department of Radiation Oncology, University Hospital and Medical Faculty, Eberhard Karls University Tübingen, Tübingen, Germany; ^2^ Section for Biomedical Physics, Department of Radiation Oncology, University Hospital and Medical Faculty, Eberhard Karls University Tübingen, Tübingen, Germany; ^3^ Department of Internal Medicine I, University Hospital Tübingen, Tübingen, Germany; ^4^ Department of General, Visceral and Transplant Surgery, University Hospital Tübingen, Tübingen, Germany; ^5^ Department of Internal Medicine I, Ulm University Hospital Medical Center, Ulm, Germany; ^6^ Department of Diagnostic and Interventional Radiology , University of Tübingen, Tübingen, Germany; ^7^ German Cancer Consortium (DKTK), partner site Tübingen; and German Cancer Research Center (DKFZ), Heidelberg, Germany; ^8^ Department of Radiation Oncology, Berlin Institute of Health, Charité - Universitätsmedizin Berlin, Corporate Member of Freie Universität Berlin, Humboldt-Universität zu Berlin, Berlin, Germany

**Keywords:** magnetic resonance guided radiotherapy, stereotactic body radiation therapy, image guided radiation therapy, liver metastases, online adaptive radiation therapy

## Abstract

**Introduction:**

Stereotactic body radiotherapy (SBRT) is used to treat liver metastases with the intention of ablation. High local control rates were shown. Magnetic resonance imaging guided radiotherapy (MRgRT) provides the opportunity of a marker-less liver SBRT treatment due to the high soft tissue contrast. We report herein on one of the largest cohorts of patients treated with online MRgRT of liver metastases focusing on oncological outcome, toxicity, patient reported outcome measures (PROMs), quality of life.

**Material and methods:**

Patients treated for liver metastases with online MR-guided SBRT at a 1,5 T MR-Linac (Unity, Elekta, Crawley, UK) between March 2019 and December 2021 were included in this prospective study. UK SABR guidelines were used for organs at risk constraints. Oncological endpoints such as survival parameters (overall survival, progression-free survival) and local control as well as patient reported acceptance and quality of life data (EORTC QLQ-C30 questionnaire) were assessed. For toxicity scoring the Common Toxicity Criteria Version 5 were used.

**Results:**

A total of 51 patients with 74 metastases were treated with a median of five fractions. The median applied BED GTV D98 was 84,1 Gy. Median follow-up was 15 months. Local control of the irradiated liver metastasis after 12 months was 89,6%, local control of the liver was 40,3%. Overall survival (OS) after 12 months was 85.1%. Progression free survival (PFS) after 12 months was 22,4%. Local control of the irradiated liver lesion was 100% after three years when a BED ≥100 Gy was reached. The number of treated lesions did not impact local control neither of the treated or of the hepatic control. Patient acceptance of online MRgSBRT was high. There were no acute grade ≥ 3 toxicities. Quality of life data showed no significant difference comparing baseline and follow-up data.

**Conclusion:**

Online MR guided radiotherapy is a noninvasive, well-tolerated and effective treatment for liver metastases. Further prospective trials with the goal to define patients who actually benefit most from an online adaptive workflow are currently ongoing.

## Introduction

With the advent of oligometastatic disease as a third disease state between “metastatic” and “non-metastatic”, there is in growing interest in effective local treatment options such as microwave ablation, surgery or radiofrequency ablation (1, 2). Stereotactic body radiotherapy (SBRT) in particular has recently been shown to prolong overall survival in patients with oligometastatic disease (3, 4). However using SBRT in the abdominal compartment and in specifically in the liver is challenging due to the very limited soft tissue contrast of cone-beam computed tomography (CBCT) based linear accelerators. For this reason fiducial markers are often used as surrogate markers. Recently online adaptive magnetic resonance tomography guided radiotherapy (MRgRT) was introduced into the clinical routine (5–9). MRgRT provides higher soft tissue contrast of MR imaging than cone-beam computed tomography (CBCT). It also allows online plan adaptation for each radiotherapy fraction (10). Especially for treatment of tumors in the abdomen the better soft tissue contrast of MR imaging allows to visualize tumors and organs at risk (OAR) at the timepoint of treatment (11). MRgRT also offers the opportunity of a marker-less SBRT without the possible complications due to the invasive fiducial placement potentially increasing patient acceptance compared to invasive procedures (12). In this study we report the largest cohorts of patients treated with online MRgRT of liver metastases focusing on oncological outcome, toxicity, patient reported outcome measures (PROMs) and quality of life.

## Materials and methods

### Patient selection

In this study consecutive patients with liver metastases receiving online MR-guided SBRT with a fraction size above 5 Gy at a 1,5 T MR-Linac (Unity, Elekta, Crawley, UK) were included. The MR-01 study (NCT04172753) is a prospective phase 2 basket trial primarily assessing the feasibility of online adaptive MR guided radiotherapy but also oncological endpoints such as survival parameters and patient-reported outcomes (PROMs). Written informed consent of all patients was provided. Prior to radiotherapy therapeutic alternatives were debated in a multidisciplinary tumor board. The institutional review board of the medical faculty Tübingen (IRB 659/2017BO1) approved the study.

### Treatment planning and radiotherapy workflow

Detailed report of the treatment planning and online workflow has been published (11). For treatment simulation and for every fraction patients had to feast for 3 hours. Patients received a four dimensional CT simulation scan in treatment position with indexed patient positioning aids. On the same day an MR simulation scan was performed on the 1.5T MR-Linac. Three MR simulation scans were performed: A triggered T2 (voxel size 2 mm × 2 mm × 2.4 mm, TE 206 ms, TR 2100 ms) and T2 spair (voxel size 2 mm × 2 mm × 2.4 mm, TE 248 ms, TR 2100 ms), both in exhale position and non– triggered T2 (voxel size 2 mm × 2 mm × 2.4 mm, TE 206 ms, TR 2100 ms).

For delineation and treatment planning Monaco ^®^ V.5.4 was used. Combining information of all available images an internal target volume was created. Information of the 4D CT as well as the cine MR images was used to determine the respiratory motion of the metastases. To account for intrafractional variablity a planning target volume (PTV) margin of three to six millimeters was added on the discretion of the treating physician. UK SABR guidelines were used for organs at risk constraints (10, 13). In case OARs constraints could not be met the encompassing dose to the PTV was lowered. BED was calculated as reported previously (12).

Depending on target localisation eight to eleven individual beam angles have been used, avoiding high-density couch structures. Plan calculation was done on the exhale phase of the four dimensional planning CT.

The workflow for SBRT application was as the following: a free breathing T2 scan (voxel size 2 mm × 2 mm × 2.4 mm, TE 206 ms, TR 2100 ms) was performed after patient positioning. A rigid registration of the daily MR to the planning CT to the was performed in the online treatment system (Monaco ^®^, Elekta AB, Stockholm, Sweden) by the attending physician. Adaptation was done by the “adapt to position” workflow to account for internal shifts and a new plan was optimized online (14). After evaluation of the adapted plan by the treating physician and after a secondary dose calculation as an online quality assurance (QA)-check, plan was approved and the treatment was initiated. Cine MR imaging with a predefined structure (usually the PTV) at a frequency of 5 Hz was performed during beam on to ensure target coverage. For QA another free breathing T2 scan was acquired post-treatment. Additional images such as diffusion weighted imaging for research purposes could be taken hereafter (15).

Beam on time and in room time (in minutes) were assessed by radiotherapy therapists. For scoring acute and late toxicity Common Toxicity Criteria Version 5 have been used. During follow- up patients were contacted by phone or seen in person. In general, the first follow-up was three months after radiotherapy and included an MRI using contrast agent, blood test, PROMs and assessment of toxicity. Afterwards follow up was repeated every 3 month.

Prior to radiotherapy blood work with liver function tests and a clinical assessment for cirrhotic liver disease (Child-Pugh score) was done. Time to event data was calculated according to the Kaplan-Meier method. For group comparisons the log-rank test was performed. Local control was calculated from the day of the last radiotherapy fraction until the first report of disease progression on imaging or histological confirmation of disease recurrence or persistence. Progression-free survival was calculated from the last radiotherapy fraction until local or distant disease progression or death of any cause. Overall survival was calculated from the last radiotherapy fraction until death of any cause. Statistics were performed using SPSS, Version 28, IBM, Armonk, New York, and Graphpad Prism 5. A p-value of less than 0.05 was considered statistically significant.

Patient reported acceptance of online MRgSBRT was assessed by a previously published questionnaire (13, 14). For radiation induced liver disease (RILD) the definition of Lawrence et al. was used (16).

## Results

### Patient and treatment characteristics

Between March 2019 and December 2021 a total of 51 consecutive patients have been treated with online MR-guided SBRT for liver metastases. Patient characteristics are shown in **Table 1**.

**Table 1:** Patient and treatment characteristics.

	n (%)
Patients	51
Sex	
Male Female	32 (62,7) 19 (37,3)
Median age (range)	67 (42 – 90)
Irradiated metastases	74
Treated metastases	
n=1 n>1	45 (78,9) 12 (21,1)
Number of hepatic metastasis prior to RT	
n=1 n>1 maximum median	31 (54,4) 26 (45,6) 4 1,7
Indication	
Oligometastatic disease Oligoprogression	43 (75,4) 14 (24,6)
Extrahepatic tumour	
Yes No	26 (45,6) 31 (54,4)
Median fractions (range)	5 (3 – 8)
Primary tumor	
Cholangiocarcinoma Colorectal Breast Choroidal melanoma Other*	7 (13,7) 23 (45,1) 2 (3,9) 4 (7,8) 15 (29,4)
Chemotherapy prior to RT	
Yes No	42 (82,4) 9 (17,6)
Previous liver directed therapy (treated lesion)	
No Yes Surgery TACE RFA SIRT Chemosaturation	39 (68,4) 18 (31,6) 13 0 1 0 4
Previous hepatic therapy (other lesions)	
No Yes Surgery TACE RFA Radiotherapy SIRT Chemosaturation	33 (57,9) 24 (42,1) 17 0 4 2 2 3
Liver cirrhosis prior to RT	
No Child Pugh A Child Pugh B Child Pugh C	51 (89,5) 5 (8,8) 1 (1,8) 0 (0)
Median chemotherapy-free time after RT (range)	4,9 (0 – 24) months
Median in room time (range)	35,7 (22,2 – 44,8) minutes
Median beam on time (range)	7,4 (4 – 12) minutes

*Esophageal cancer (n=2), gastrointestinal stromal tumor (GIST, n=2), pancreas (adenocarcinoma) (n=2), n=1 for esophagus, adenoidcystic carcinoma of the head and neck, renal cell carcinoma, epipharyngeal cancer, ovarial cancer, yolk sac tumor, neuroendocrine tumor (NET) of the pancreas, NET of the small bowel, adenocarcinoma of unknown primary site.

TACE (Transcatheter arterial chemoembolization), RFA (Radiofrequency ablation), SIRT (Selective internal radiation therapy).

Median patient age was 67 years (range 42 – 90 years). Of the 51 patients, 24 patients (47%) had received liver directed local treatment prior to SBRT and 42 (82,4%) of the patients had received chemotherapy. In 45 patients a single lesion was treated, 12 patients received treatment of more than one liver lesion using separate treatment plans.

Dosimetric parameters are summarized in **Table 2**.

**Table 2:** Dosimetric parameters. GTV-Gross tumor volume, IQR-Inter quartile range.

	median	minimal	maximal	25% quartile	75% quartile	IQR
**ITV volume (cc)**	23,4	0,5	201,4	4,5	27,8	23,3
**PTV volume (cc)**	48,9	3,0	260,5	13,0	71,7	58,7
**Liver volume (cc)**	1432,8	852,7	3011,1	1129,1	1633,3	504,2
**Liver minus GTV volume (cc)**	1451,1	873,6	3056,7	1156,6	1642,9	486,3
**Mean dose liver minus GTV (Gy)**	7,1	0,6	12,9	4,8	9,9	5,1
**Mean dose GTV (Gy)**	47,1	22,2	62,1	40,5	53,1	12,7
**Maximum dose GTV (Gy)**	50,3	26,4	67,8	42,2	57,7	15,5
**GTV D98% (Gy)**	43,7	19,3	55,7	38,6	49,8	11,2

GTV, Gross Tumor Volume; ITV, Internal Target Volume; PTV, Planning Target Volume; IQR, inter-quartile range.

A median of five fractions were applied (range three to eight fractions). Median beam-on time was 7,4 min (4 -12 range). The median in room time was 35,56 min (22,2 – 44,8).

The median applied BED GTV D98 was 84,1 Gy (26,7 – 135,5 Gy). The median applied BED ITV D98 was 81,4 Gy (29,1 – 132,9 Gy).

There were no acute grade ≥ 3 toxicities. No change in Child-Pugh Score was observed during follow-up.

### Oncological outcome

Median follow-up was 15 months (3 – 39 months). Median chemotherapy-free interval after completion of SBRT was 4.9 months (0 – 24 months) after SBRT.

**Figure 1:**
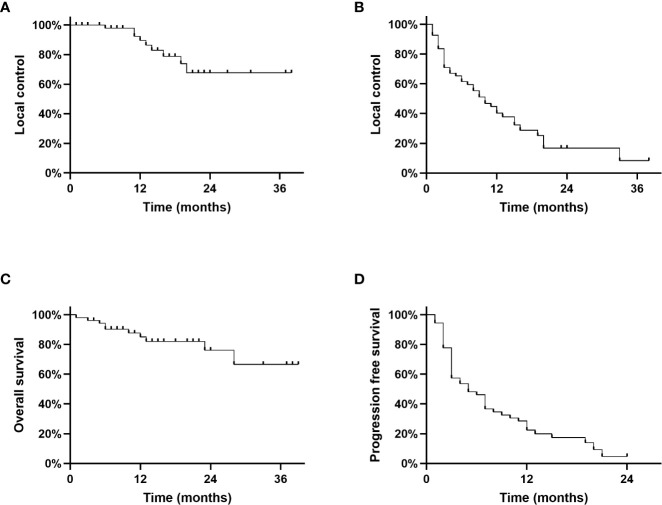
**(A)**: Local control of the irradiated liver lesion, **(B)**: Local control rate of the liver, **(C)**: Overall survival, **(D)**: Progression free survival.

Local control of the irradiated liver metastasis after 12 months was 89,6%; after 24 months 67,7% and after 36 months 67,7% (**Figure 1A**). Local control of the liver, outside of the irradiated liver lesion was 40,3% after 12 months, 16,8% after 24 months and 8,4% after 36 months as shown in **Figure 1B**. Overall survival after 12 months, 24 months and 36 months were 85.1%, 76.2% and 66.7%. Median OS was not reached (**Figure 1C**). Progression free survival (PFS) after 12 months was 22,4% and 4.7% after 24 months. Median PFS was 5 months (**Figure 1D**).

No difference in local control regarding the irradiated lesion was observed between metastasis originating from colorectal vs non-colorectal primary sites (p=0.64), **Supplementary figure 1**.

**Figure 2:**
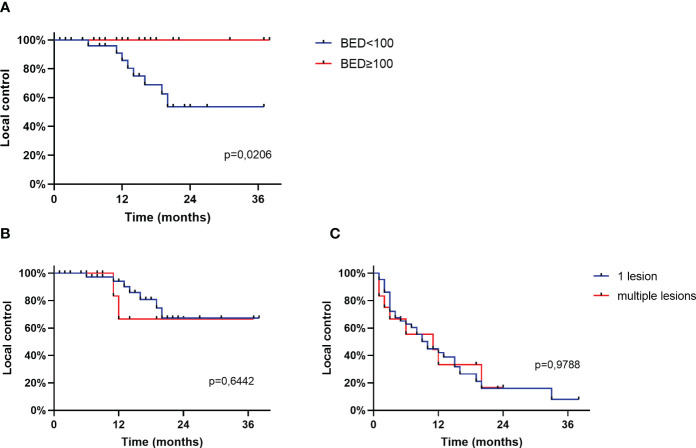
**(A)**: Local control of the irradiated liver lesion based on BED, **(B)**: Local control of the irradiated liver lesion based on number of treated lesions, **(C)**: Local control of the liver based on number of treated lesions.

Local control of the irradiated liver lesion was 100% after three years when a BED ≥100 Gy was reached and 85.7%, 53.6% and 53.6% after 12, 24 and 36 months respectively, when a BED < 100 Gy was applied (p=0,02) as shown in **Figure 2A**. The number of treated lesions did not impact local control neither of the treated lesions (66,7% vs 66,7% after 24 months) or of the hepatic control (16,7% vs 16,7% after 24 months) as shown in **Figures 2B, C**. Local control when a single lesion was treated was 77,4% after 12 months (24 months: 37,1%, 36 months: 37,1%). After treatment of multiple liver lesions local control was 55,0% after 12 months (24 months: 55,0%, 36 months: 55,0%).

**Figure 3:**
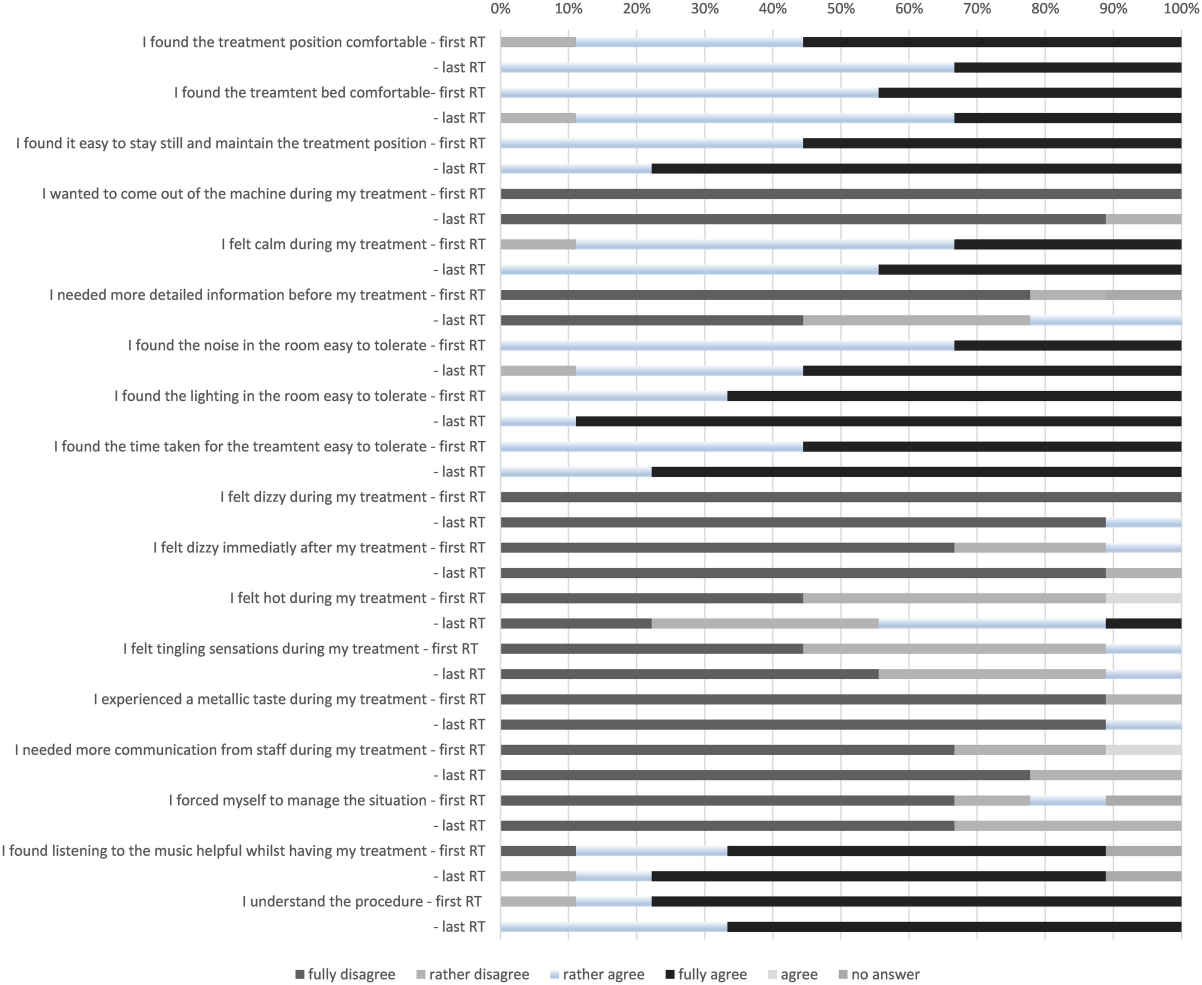
Patient acceptance of various aspects of online MRgSBRT. RT (radiotherapy).

Patient acceptance of online MRgSBRT was high as shown in **Figure 3**.

**Figure 4:**
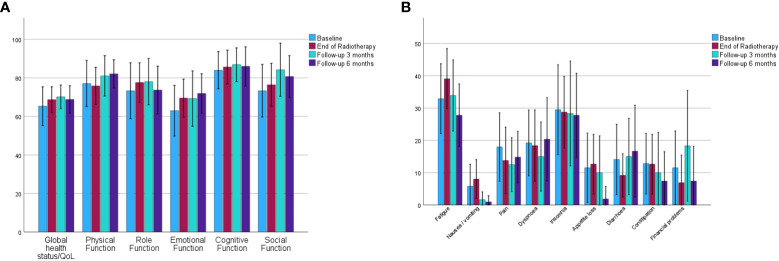
Mean values and 95% confidence intervals for EORTC QLQ-C30 data for global health status and function subscales **(A)** and symptom subscales **(B)**. (EORTC QLQ-C30, European Organisation for Research and Treatment of Cancer core questionnaire).

Quality of life data assessed by the EORTC QLQ-C30 questionnaire is shown in **Figures 4A, B**.

Quality of life data was available before the start of radiotherapy (26 patients), at last radiotherapy (29 patients), at three months follow-up (20 patients) and at six months follow-up (19 patients). All comparisons between baseline and “last radiotherapy fraction”, “3 months follow-up” and “6 months follow-up” showed no significant difference, apart from “appetite loss” being significantly lower at six months follow-up compared with baseline (11.5 vs. 1.8, p=0.04).

## Discussion

With the “introduction” of the oligometastatic disease state as a third state between non-metastastic and diffusely metastatic and the associated paradigm shift towards local metastases directed therapies there is growing need for effective and non-invasive local treatments for patients presenting with oligometastases (3, 4).

The present study reports the largest cohort of liver metastases treated on a 1.5 T MR-Linac. We had previously published data on the feasibility of the online workflow and the imaging quality with an excellent visibility of the majority of the lesions treated (11). As in our previous report patient acceptance of the treatment was excellent and no treatment had to be discontinued due to patient request. This is reassuring as there had been concerns initially whether patients could manage to remain still in an MRI with arms above head for the duration of treatment. Data on treatment outcomes after online-MR guided radiotherapy for liver metastases is still sparse.

A selection of studies on MR guided stereotactic body radiotherapy of liver metastases is shown in **Table 3**.

**Table 3:** Studies on MR guided stereotactic body radiotherapy of liver metastases reporting local control and survival data. (OS – Overall survival, LC – local control, PFS – progression free survival).

Author	Year	Primary or secondary tumors	Patients (n)	Patients with liver metastases (n)	Patients with primary tumors (n)	Median Dose	Median fraction	OS	LC
Van Dams et al.	2022	Primary liver tumors, liver metastases	20	12	8	54 Gy (11,5-60)	3 (1-5)	2 year: 50,7%	1 year: 94,7% 2 year: 79,6%
Ugurluer et al.	2021	Liver metastases	21	21	0	50 Gy (40-60)	5 (3-8)	1 year: 93% 2 year: 93%	1 year: 89,7%, 2 year: 64,6% (intrahepatic PFS)
Yoon et al.	2021	Primary and metastatic tumors (abdomen, pelvis)	106	46	60	40 Gy (24-60)	5 (3-5)	1 year: 79%2 year: 57%	1 year: 87% 2 year: 71%
Weykamp et al.	2021	Liver metastases, HCC	20	18	2	50 Gy (45-60)	8 (3-12)	1 year: 84%	1 year: 88,1%
Rosenberg et al.	2019	Primary liver tumors, liver metastases	26	18	8	50 Gy	5	2 year: 60%	21,2 months: 80,4%
Henke et al.	2017	Primary liver tumors, liver metastases, other abdominal sites	20	5	10	50 Gy	5	1 year: 75%	15 months: 90%

For instance Weykamp and colleagues report a one year local control rate of 88% in twenty patients treated for liver tumors (18 metastases, two HCCs) on a 0.35 T MR-Linac (17). Van Dams et al. also report data of a mixed cohort (n=20) of eight patients with primary and 12 patients with secondary liver tumors (18). In that study, one and two year local were 94.7% and 79.6%, respectively. Ugurluer et al. reported an intra- and extrahepatic progression-free survival of 89.7% and 73.5% after one year in 21 oligometastatic patients and a 1-year overall survival of 93.3% (19). Yoon et al. retrospectively analyzed SBRT of Primary and metastatic tumors and reported a local control after 1 year of 87% an after 2 years 71%. In case of lesions treated with BED >=100 a local control after 2 years of 96% was shown (20).

While the actual adaptive workflow in the treatment with adaptive radiotherapy is the same independent of the underlying histology, the indication for treatment, comorbidities, competing risks and radiosensitivity are different between primary and secondary liver tumors. We have therefore opted to report outcomes for liver metastases exclusively. With a one year and three local control rate of approximately 90% and 70% respectively our results are favorable in particular since lower local control rates have been reported for liver metastases compared with primary liver tumors before (21). Local control rates for liver metastases after treatment on cone-beam CT based linear accelerators vary in the literature (22–24). Using MR guidance we were able to omit the placement of fiducial markers and facilitate a fully non-invasive workflow. Furthermore as in our previous report, we were able to visualize almost all tumors and therefore ensure adequate tumor coverage (11). When interpreting our results it has to be considered that most patients were heavily pretreated systemically and often have had other local liver directed treatments before being referred for radiotherapy. We observed the strong impact of the biological effective dose on the local control of the treated metastases with 100% local control in lesions that were treated with a BED of 100 Gy or more. This is in line with results from previous reports (18, 25). The question may arise while patients are treated with a BED of less than 100 Gy. The decision to prescribe a BED below or higher than 100 Gy is always driven by the present clinical scenario. Patients with oligometastatic disease are more likely to receive higher doses potentially accepting a higher likelihood for normal tissue complications than patients to were treated for oligoprogressive disease when the sole goal of treatment is to prolong the interval without systemic treatment or maintenance of the systemic treatment that is well-tolerated (26). Very few reports have longitudinally assessed quality of life and symptom scores in patients who have received stereotactic radiotherapy for liver metastases (27). In our cohort using the EORTC QLQ-C30 questionnaire we observed widely stable scores for quality of life and symptom scales holding true comparing both the time from baseline to the last fraction of radiotherapy and also during a six-month follow-up. This can likely be explained by the precise treatment and the median chemotherapy free interval of five months observed over all patients. The strength of our study lies in its sample size and prospective character assuring stringent follow-up using regular imaging studies and the standardized assessment of quality of life and toxicity. Despite including only patients who were treated for metastases, there is a heterogeneity in terms of the underlying primary tumors which is a limitation. When we conducted this trial the 1.5 Tesla MR-Linac did not support a gated treatment. Using a gated workflow tumors can be irradiated in a predefined position during the respiratory cycle resulting in the smallest possible volume to be treated at the price of a longer treatment time per fraction (17). However, motion management strategies have recently been announced also for the 1.5 Tesla MR-Linac.

## Conclusion

Online MR guided radiotherapy is a noninvasive, well-tolerated and effective treatment for liver metastases. Further prospective trials with the goal to define patients who actually benefit most from an online adaptive workflow are currently ongoing (28).

## Data availability statement

The raw data supporting the conclusions of this article will be made available by the authors, without undue reservation.

## Ethics statement

The studies involving human participants were reviewed and approved by the institutional review board of the medical faculty Tübingen (IRB 659/2017BO1). The patients/participants provided their written informed consent to participate in this study.

## Author contributions

Conceptualization: LU, CG, SBo, DZ. Data analysis: LU, CG, SBo. Writing original draft preparation: LU, CG, SBo, DZ. Writing, review and editing: LU, CG, SBo. All authors contributed to the article and approved the submitted version

## References

[1] Al Bandar MH Kim NK. Current status and future perspectives on treatment of liver metastasis in colorectal cancer (Review). Oncol Rep (2017) 37(5):2553–64. doi: 10.3892/or.2017.5531 28350137

[2] Jackson WC Tao Y Mendiratta-Lala M Bazzi L Wahl DR Schipper MJ. Comparison of stereotactic body radiation therapy and radiofrequency ablation in the treatment of intrahepatic metastases. Int J Radiat Oncol Biol Phys (2018) 100(4):950–8. doi: 10.1016/j.ijrobp.2017.12.014 PMC6142177 29485074

[3] Gomez DR Blumenschein GR Jr. Lee JJ Hernandez M Ye R Camidge DR. Local consolidative therapy versus maintenance therapy or observation for patients with oligometastatic non-small-cell lung cancer without progression after first-line systemic therapy: A multicentre, randomised, controlled, phase 2 study. Lancet Oncol (2016) 17(12):1672–82. doi: 10.1016/S1470-2045(16)30532-0 PMC5143183 27789196

[4] Palma DA Olson R Harrow S Gaede S Louie AV Haasbeek C. Stereotactic ablative radiotherapy versus standard of care palliative treatment in patients with oligometastatic cancers (SABR-COMET): A randomised, phase 2, open-label trial. Lancet (2019) 393(10185):2051–8. doi: 10.1016/S0140-6736(18)32487-5 30982687

[5] Kluter S. Technical design and concept of a 0.35 T MR-linac. Clin Transl Radiat Oncol (2019) 18:98–101. doi: 10.1016/j.ctro.2019.04.007 31341983 PMC6630153

[6] Winkel D Bol GH Kroon PS van Asselen B Hackett SS Werensteijn-Honingh AM. Adaptive radiotherapy: The elekta unity MR-linac concept. Clin Transl Radiat Oncol (2019) 18:54–9. doi: 10.1016/j.ctro.2019.04.001 PMC6630157 31341976

[7] Boldrini L Corradini S Gani C Henke L Hosni A Romano A. MR-guided radiotherapy for liver malignancies. Front Oncol (2021) 11:616027. doi: 10.3389/fonc.2021.616027 33869001 PMC8047407

[8] Nachbar M Monnich D Kalwa P Zips D Thorwarth D Gani C. Comparison of treatment plans for a high-field MRI-linac and a conventional linac for esophageal cancer. Strahlenther Onkol (2019) 195(4):327–34. doi: 10.1007/s00066-018-1386-z 30361744

[9] Nachbar M Monnich D Boeke S Gani C Weidner N Heinrich V. Partial breast irradiation with the 1.5 T MR-linac: First patient treatment and analysis of electron return and stream effects. Radiother Oncol (2020) 145:30–5. doi: 10.1016/j.radonc.2019.11.025 31874347

[10] Hanna GG Murray L Patel R Jain S Aitken KL Franks KN. UK Consensus on normal tissue dose constraints for stereotactic radiotherapy. Clin Oncol (R Coll Radiol) (2018) 30(1):5–14. doi: 10.1016/j.clon.2017.09.007 29033164

[11] Gani C Boeke S McNair H Ehlers J Nachbar M Mönnich D. Marker-less online MR-guided stereotactic body radiotherapy of liver metastases at a 1.5 T MR-linac - feasibility, workflow data and patient acceptance. Clin Transl Radiat Oncol (2021) 26:55–61. doi: 10.1016/j.ctro.2020.11.014 33319073 PMC7723999

[12] Fowler JF. 21 years of biologically effective dose. Br J Radiol (2010) 83(991):554–68. doi: 10.1259/bjr/31372149 PMC3473681 20603408

[13] Barnes H Alexander S Bower L Ehlers J Gani C Herbert T. Development and results of a patient-reported treatment experience questionnaire on a 1.5 T MR-linac. Clin Transl Radiat Oncol (2021) 30:31–7. doi: 10.1016/j.ctro.2021.06.003 PMC8283148 34307911

[14] Olausson K Holst Hansson A Zackrisson B Edvardsson D Östlund U Nyholm T. Development and psychometric testing of an instrument to measure the patient's experience of external radiotherapy: The radiotherapy experience questionnaire (RTEQ). Tech Innov Patient Support Radiat Oncol (2017) 3-4:7–12. doi: 10.1016/j.tipsro.2017.06.003 32095560 PMC7033812

[15] Leibfarth S Winter RM Lyng H Zips D Thorwarth D. Potentials and challenges of diffusion-weighted magnetic resonance imaging in radiotherapy. Clin Transl Radiat Oncol (2018) 13:29–37. doi: 10.1016/j.ctro.2018.09.002 30294681 PMC6169338

[16] Lawrence TS Robertson JM Anscher MS Jirtle RL Ensminger WD Fajardo LF. Hepatic toxicity resulting from cancer treatment. Int J Radiat Oncol Biol Phys (1995) 31(5):1237–48. doi: 10.1016/0360-3016(94)00418-K 7713785

[17] Weykamp F Hoegen P Klüter S Spindeldreier CK König L Seidensaal K. Magnetic resonance-guided stereotactic body radiotherapy of liver tumors: Initial clinical experience and patient-reported outcomes. Front Oncol (2021) 11:610637. doi: 10.3389/fonc.2021.610637 34178616 PMC8219972

[18] van Dams R Wu TC Kishan AU Raldow AC Chu FI Hernandez J. Ablative radiotherapy for liver tumors using stereotactic MRI-guidance: A prospective phase I trial. Radiother Oncol (2022) 170:14–20. doi: 10.1016/j.radonc.2021.06.005 34107296

[19] Ugurluer G Mustafayev TZ Gungor G Atalar B Abacioglu U Sengoz M. Stereotactic MR-guided online adaptive radiation therapy (SMART) for the treatment of liver metastases in oligometastatic patients: Initial clinical experience. Radiat Oncol J (2021) 39(1):33–40. doi: 10.3857/roj.2020.00976 33794572 PMC8024184

[20] Yoon SM Luterstein E Chu FI Cao M Lamb J Agazaryan N. Clinical outcomes of stereotactic magnetic resonance image-guided adaptive radiotherapy for primary and metastatic tumors in the abdomen and pelvis. Cancer Med (2021) 10(17):5897–906. doi: 10.1002/cam4.4139 PMC8419771 34288538

[21] Ohri N Tomé WA Méndez Romero A Miften M Ten Haken RK Dawson LA. Local control after stereotactic body radiation therapy for liver tumors. Int J Radiat Oncol Biol Phys (2021) 110(1):188–95. doi: 10.1016/j.ijrobp.2017.12.288 PMC6102100 29395629

[22] Andratschke N Alheid H Allgäuer M Becker G Blanck O Boda-Heggemann J. The SBRT database initiative of the German society for radiation oncology (DEGRO): Patterns of care and outcome analysis of stereotactic body radiotherapy (SBRT) for liver oligometastases in 474 patients with 623 metastases. BMC Cancer (2018) 18(1):283. doi: 10.1186/s12885-018-4191-2 29534687 PMC5851117

[23] Scorsetti M Comito T Clerici E Franzese C Tozzi A Iftode C. Phase II trial on SBRT for unresectable liver metastases: Long-term outcome and prognostic factors of survival after 5 years of follow-up. Radiat Oncol (2018) 13(1):234. doi: 10.1186/s13014-018-1185-9 30477560 PMC6258482

[24] Dawood O Mahadevan A Goodman KA. Stereotactic body radiation therapy for liver metastases. Eur J Cancer (2009) 45(17):2947–59. doi: 10.1016/j.ejca.2009.08.011 19773153

[25] Joo JH Park J-h Kim JC Yu CS Lim S-B Park IJ. Local control outcomes using stereotactic body radiation therapy for liver metastases from colorectal cancer. Int J Radiat Oncol Biol Physics (2017) 99(4):876–83. doi: 10.1016/j.ijrobp.2017.07.030 29063852

[26] Guckenberger M Lievens Y Bouma AB Collette L Dekker A deSouza NM. Characterisation and classification of oligometastatic disease: A European society for radiotherapy and oncology and European organisation for research and treatment of cancer consensus recommendation. Lancet Oncol (2020) 21(1):e18–28. doi: 10.1016/S1470-2045(19)30718-1 31908301

[27] Méndez Romero A Wunderink W van Os RM Nowak PJCM Heijmen BJM Nuyttens JJ. Quality of life after stereotactic body radiation therapy for primary and metastatic liver tumors. Int J Radiat Oncol Biol Physics (2008) 70(5):1447–52. doi: 10.1016/j.ijrobp.2007.08.058 17996394

[28] Regnery S Ristau J Weykamp F Hoegen P Sprengel SD Paul KM. Magnetic resonance guided adaptive stereotactic body radiotherapy for lung tumors in ultracentral location: The MAGELLAN trial (ARO 2021-3). Radiat Oncol (2022) 17(1):102. doi: 10.1186/s13014-022-02070-x 35614486 PMC9134672

