# Supplementary material for: Local control and patient reported outcomes after online MR guided stereotactic body radiotherapy of liver metastases
Source: Front Oncol. 2023 Jan 16;12:1095633. doi: 10.3389/fonc.2022.1095633 (PMC9885175; doi:10.3389/fonc.2022.1095633)
Supplement: Supplementary file 1 [file DataSheet_1.docx]

**Supplementary Material**

No difference in local control regarding the irradiated lesion was observed between metastasis originating from colorectal vs non-colorectal primary sites (p=0.64), supplementary Figure 1).

**
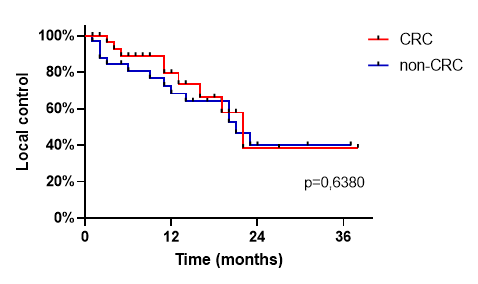
**

**Supplementary Figure 1: Local control of the irradiated liver lesion sorted by metastasis originating from colorectal (CRC) vs non-colorectal (non-CRC) primary sites**
